# Auto-antibodies against type I IFNs are associated with severe COVID-19 pneumonia

**DOI:** 10.1038/s41392-021-00514-6

**Published:** 2021-02-26

**Authors:** Weilin Zhou, Wei Wang

**Affiliations:** State Key Laboratory of Biotherapy and Cancer Center, West China Hospital, Sichuan University, and Collaborative Innovation Center for Biotherapy, Chengdu, People’s Republic of China

**Keywords:** Infectious diseases, Infectious diseases

**Fig. 1:**
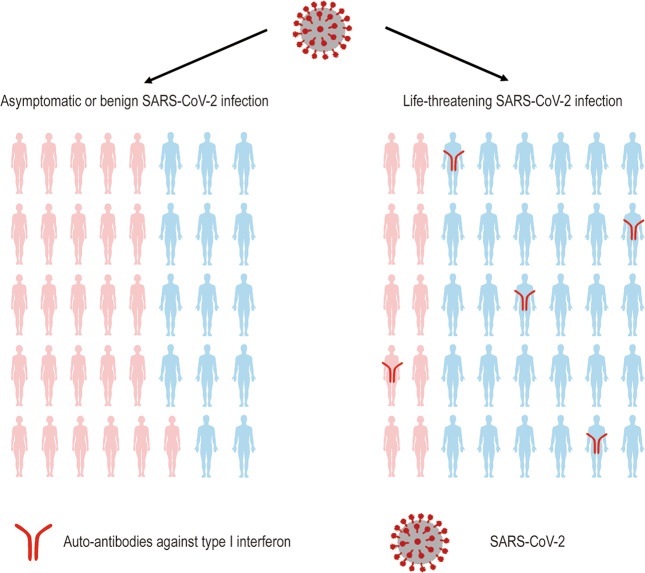
Patients with life-threatening COVID-19 pneumonia caused by the SARS-CoV-2 virus produce auto-Abs (red) against type 1 IFNs.^1^

A recent work published in Science by Paul Bastard et al.^1^ reported that neutralizing auto-antibodies (auto-Abs) against type I interferons (IFNs) were distinctly found in patients with life-threatening COVID-19 pneumonia (101/987, 10.2%), while were rarely detected in asymptomatic, benign infectious, or healthy individuals (Fig. 1). Among the patients with severe COVID-19 pneumonia who have neutralizing auto-Abs against type I IFNs, male patients account for the vast majority (95/101, 94%), suggesting that pathogenic gene encoding auto-Abs may be linked to gender.

The COVID-19 pandemic caused by SARS-CoV-2 has caused more than one million mortality worldwide. The clinical manifestations of patients vary greatly, ranging from asymptomatic infection to fatal disease. Jérôme Hadjadj et al. reported that deficiency of type I IFNs could be a hallmark of severe COVID-19.^2^ The type I interferon serves as an alarm for the human to combat viral infection. It can effectively contribute to innate immunity and cell-intrinsic immunity when the body is invaded by viruses or bacteria. Therefore, whether type 1 interferon can function properly will directly affect the progression of COVID-19 pneumonia. In another recent study published in Science, Qian Zhang et al.^3^ reported that 3.5% (23 of 659) of patients with life-threatening COVID-19 had inborn errors in 13 human loci. The aforementioned antiviral defense loci govern TLR3- and IRF7-dependent type I IFN immunity to influenza virus, suggesting that inborn type I IFNs-related gene defects may result in progression of COVID-19 pneumonia. In addition to congenital errors, Paul Bastard et al. noticed that neutralizing IgG auto-Abs against type I IFNs exist in three patients with autoimmune polyendocrinopathy syndrome type I (APS-1) before infection of SARS-CoV-2^4^ and they all developed into life-threatening COVID-19 pneumonia. Hence, researchers put forward a hypothesis that perhaps the life-threatening COVID-19 pneumonia might also be ascribed to the neutralizing auto-Abs against type I IFNs.

In order to verify this conjecture, Paul Bastard et al. used multiple methods (multiplex particle-based flow cytometry, Enzyme-linked immunosorbent assay, and Luminex technology) to detect the levels of auto-Abs in plasma or serum samples from three groups with different stages of disease. The results showed that 135 patients with critical COVID-19 had high levels of the IgG auto-Abs against IFN-α2 and/or IFN-ω in plasma or serum. Among them, 49 patients were positive for Abs against both IFN-α2 and IFN-ω, 45 were positive for Abs against both IFN-α2, 41 were positive for Abs against IFN-ω. Conversely, no auto-Abs were detected in patients from the mild or asymptomatic groups and four patients in the healthy population were detected with auto-Abs. In brief, compared to asymptomatic or mild patients and healthy individuals, the phenomenon that 13.7% of patients with life-threatening COVID-19 pneumonia had IgG auto-Abs against at least one type I IFN is an obvious distinction. Interestingly, 15 patients with life-threatening COVID-19 not only had auto-Abs against IFN-α2 and/or IFN-ω, but also had auto-Abs against other cytokines (IFN-γ, GMCSF, IL-6, IL-10, IL-12p70, IL-22, IL-17A, IL-17F, and/or TNFβ). Although the patients had auto-Abs that bind to multiple cytokines, the researchers observed that only 3 cytokines (IL-12p70, IL-22, IL-6) in some patients were completely neutralized by auto-Abs. The finding demonstrates that not all auto-Abs possess the capacity to neutralized cytokines. Whereafter, researchers tested the neutralizing ability of auto-Abs against IFN-α2 and IFN-ω in vitro. By co-incubating PBMCs from healthy donors with IFN-α2 and/or IFN-ω in the presence of plasma derived from patients with auto-Abs, they observed that STAT1 phosphorylations of 101 patients were abolished completely. Furthermore, by analyzing the induction of ISG CXCL10, the researchers found that auto-Abs effectively block the function of IFN-α2 in vitro, but the function of IFN-γ was still valid. Later, researchers further explored the diversity of auto-Abs by means of multiple methods. Among the 22 selected subjects (who had been confirmed to have auto-Abs against IFN-α2), all patients had auto-Abs against all 13 subtypes of IFN-α, 2 patients had auto-Abs to attack IFN-β, 2 had auto-Abs against IFN-ε and one against IFN-κ. They proved that the effect of IFN-α2 in preventing the SARS-CoV-2 from infecting Huh7.5 cells was eliminated by the serum containing auto-Abs against type I IFNs. Furthermore, they found that low (1 patient) or undetectable (40 patients) levels of the 13 types of IFN-α were tested in plasma of 41 patients with auto-Abs. These results indicated that auto-Abs have the potential to increase the infection of SARS-CoV-2 and aggravate the disease. Surprisingly, there is a marked gender bias in the group with life-threatening COVID-19 pneumonia and neutralizing auto-Abs against type I IFNs, where 95 of the 101 patients with auto-Abs for IFNs were male. Moreover, 49.5% of patients with auto-Abs were over 65 years old, suggesting that gender and aging may bring an increased frequency of auto-Abs.

Overall, Paul Bastard et al. presented multiple evidences to demonstrate that auto-Abs against type I IFNs can be a risk factor of life-threatening COVID-19 pneumonia. This research has directive significance for the clinical treatment of COVID-19 pneumonia. First, detecting auto-Abs levels can provide preventive protection in advance for patients at risk of developing severe SARS-Cov-2 pneumonia. Second, the inhibition of type I IFNs response by autoantibodies may be the key pathogenesis of COVID-19 pneumonia exacerbation, suggesting that we could alleviate the effects of the auto-Abs by plasmapheresis or compensation for type I IFNs.^5^ Finally, research also prompts us to pay attention to the dangers of auto-Abs when treating other diseases caused by viral infections. In the future, endeavors should be addressed to elucidate the reason and mechanism of auto-Abs production. Whether this is a universal risky factor in other viral infectious diseases should be answered. It is believed that searching for other defects in the immune system of patients with severe COVID-19 pneumonia will provide essential clues for the determination of clinical treatments.
